# Bright sinus appearance on arterial spin labeling MR imaging aids to identify cerebral venous thrombosis

**DOI:** 10.1097/MD.0000000000008244

**Published:** 2017-10-13

**Authors:** Ji Hee Kang, Tae Jin Yun, Roh-Eul Yoo, Byung-Woo Yoon, A Leum Lee, Koung Mi Kang, Seung Hong Choi, Ji-Hoon Kim, Chul-Ho Sohn, Moon Hee Han

**Affiliations:** a Institute of Radiation Medicine, Seoul National University Medical Research Center; b Department of Radiology; c Clinical Research Center for Stroke, Clinical Research Institute; d Department of Neurology, Seoul National University Hospital, Seoul; e Department of Radiology, Soonchunhyang University Bucheon Hospital, Gyunggi-do, Republic of Korea.

**Keywords:** arterial spin labeling, cerebral venous thrombosis, magnetic resonance imaging, perfusion MRI, venous infarct

## Abstract

Cerebral venous thrombosis is a potentially lethal disease. Early diagnosis is essential to improve its prognosis. However, its early diagnosis based on conventional imaging modalities remains a challenge in clinical settings. The purpose of this study was to evaluate whether bright sinus appearance on arterial spin-labeling perfusion-weighted image (ASL-PWI) could help identify cerebral venous thrombosis.

ASL-PWI of 13 patients who were confirmed as cerebral venous thrombosis based on neurologic symptoms and computed tomography (CT) or magnetic resonance (MR) venography (with/without cerebral angiography) were retrospectively analyzed for the presence or absence of the following: bright signal in dural sinus termed “bright sinus appearance”; and hypoperfusion in brain parenchyma drained by thrombosed sinus. In addition, conventional MR findings, including susceptibility vessel sign, empty delta sign, and atypical distribution against arterial territory, were also analyzed.

Bright sinus appearance on ASL-PWI was found in all (100%) 13 patients. In addition, 10 (77%) patients showed hypoperfusion in the brain parenchyma drained by thrombosed sinus on ASL-PWI. Susceptibility vessel sign and empty delta sign were revealed in 11 (85%) and 7 (54%) patients, respectively. Atypical distribution against arterial territory was seen in 5 (50%) of the 10 patients with parenchymal abnormality on conventional MR sequences. Therefore, the bright sinus appearance had higher sensitivities for identifying cerebral venous thrombosis than the susceptibility vessel sign, empty delta sign, and atypical distribution against arterial territory (with differences of 15%; *P* = .500, 46%; *P* = .031, and 50%; *P* = .031, respectively).

Bright sinus appearance on ASL-PWI can provide important diagnostic clue for identifying cerebral venous thrombosis. Therefore, this technique may have the potential to be used as a noninvasive diagnostic tool to identify the cerebral venous thrombosis.

## 1 Introduction

Cerebral venous thrombosis, an uncommon cerebrovascular disorder, accounts for 0.5% of all strokes.^[1]^ Even though it is a potentially lethal disease, early and prompt diagnosis with treatment such as anticoagulation and thrombolysis can improve its prognosis.^[2]^ Neurologic imaging plays major roles in its diagnosis because causal factors and clinical manifestation of cerebral venous thrombosis can vary.^[3,4]^ Even though multiple modalities including computed tomography (CT) and magnetic resonance (MR) imaging including venography have been used,^[4–6]^ proper and early diagnosis based on conventional imaging modalities remains a challenge in clinical settings. And, application of invasive imaging including digital subtraction angiography is warranted for many cases.

Recently, arterial spin-labeling perfusion-weighted image (ASL-PWI) has been incorporated as a part of the evaluation for cerebrovascular diseases in our institution. ASL-PWI is an emerging MR perfusion method for measuring cerebral blood flow (CBF) by using freely diffusible tracer. In this MR technique, the protons of arterial water in the feeding vasculature of the brain are magnetically labeled, and the labeled arterial protons then flow through the vascular tree and exchange with those in unlabeled brain tissue.^[7,8]^ A perfusion-weighted image can be acquired by subtracting image in which the inflowing arterial spins have been labeled from another image in which spin labeling has not been performed.^[7–10]^ The primary advantage of using ASL-PWI for perfusion measurement is that this technique is completely noninvasive and does not expose the patient to contrast agents.^[10]^ According to the previous reports, ASL-PWI has a potential to evaluation the cerebral perfusion status and occlusion site in arterial ischemic stroke or brain death.^[11–13]^ However, usefulness of ASL-PWI in venous infarct patients with cerebral venous thrombosis has not been elucidated. With its increasing use, we have encountered venous infarct patients with cerebral venous thrombosis showing characteristic avid bright signal in dural sinus proximal to thrombus (which we termed “bright sinus appearance”). To the best of our knowledge, the usefulness of bright sinus appearance on ASL-PWI for identifying cerebral venous thrombosis has not been elucidated yet.

Therefore, the objective of this study was to evaluate whether bright sinus appearance on ASL-PWI could be used to help identify cerebral venous thrombosis in venous infarct patients.

## 2 Materials and methods

This retrospective study was approved by the Institutional Review Board of Seoul National University Hospital. Informed consent was waived due to its retrospective nature.

### 2.1 Patients

Our electronic medical record database from between May 2010 and June 2017 was used to search for patients who were confirmed as cerebral venous thrombosis based on neurologic symptoms with suspected cerebral venous thrombosis based on CT or MR venography (with/without cerebral angiography). Among the 24 patients initially identified, 11 patients were excluded due to no ASL images (n = 9) or ASL images with poor quality, inadequate acquisition times, or artifacts (n = 2). Finally, 13 patients (7 males and 6 females) with a median age of 58 years (range, 24–78 years) were included in this study.

To compare ASL findings between cerebral venous thrombosis and arterial occlusion, we searched patients who underwent MR imaging (MRI) for suspected acute ischemic stroke or brain death from January 2017 to June 2017. Among 271 patients, those whose MR angiography demonstrated intracranial artery occlusion were included. Finally, 20 patients with arterial occlusion were selected (7 males and 13 females; median age, 71 years; age range, 34–98 years). There was no patient with brain death in the study period for control group.

### 2.2 MR imaging protocol

All patients underwent MRI on a 1.5-T unit [Signa HDxt; GE Healthcare, Milwaukee, WI (n = 5)] or 3-T units [Discovery 750; GE Healthcare (n = 3); Verio; Siemens, Erlangen, Germany (n = 1)]. MRI sequences consisted of T1-weighted image (T1WI), T2-weighted image (T2WI), fluid-attenuated inversion recovery (FLAIR), diffusion-weighted image (DWI) (b factor at 0 and 1000 mm/s^2^), susceptibility-weighted image (SWI), contrast-enhanced T1WI, and 3-dimensional time-of-flight MR venography, and ASL-PWI.

The ASL perfusion imaging was performed using a pseudo-continuous ASL pulse sequence. For first 2 MR scanners (Signa HDxt and Discovery 750; GE Healthcare), the ASL parameters were as follows: labeling pulse duration = 1.5 s, post-labeling delay = 1.5 s, TR = 4446 to 4564 ms, TE = 9.4 to 9.9 ms, field-of-view = 240 × 240 mm, number of excitations = 3, number of interleaved slices = 32, and slice thickness = 5 mm. For another MR scanner (Verio; Siemens Healthcare), ASL images were acquired with a background-suppressed 3-dimensional gradient and spin echo single-shot readout (labeling pulse duration = 1.5 s, post-labeling delay = 1.6 s, no flow crushing gradient, TR = 3660 ms, TE = 14 ms, field-of-view = 240 × 240 × 96 mm, matrix = 64 × 64 × 11, 60 pairs of tags and controls, acquired in 4 minutes, whole brain coverage). Signal intensity change between labeled and control images was fitted to a previously published model to obtain a quantitative perfusion map of CBF.^[12]^

### 2.3 Image analysis

All ASL images were visually analyzed by 2 radiologists (J.H.K. and T.J.Y. with 2 and 14 years of experience, respectively) for the presence or absence of the following: bright signal intensity in dural sinuses proximal to thrombus termed “bright sinus appearance”; hypoperfusion in brain parenchyma drained by the thrombosed sinus; and bright signal intensity in arterial segment. Three square regions of interest were placed in the least perfused areas of the lesions with area of 1 cm^2^. We calculated mean CBF values from the 3 regions of interest. The relative CBF was obtained by dividing mean CBF values of the lesion with mean CBF of 3 regions of interest in the contralateral normal-appearing brain parenchyma.

On conventional MRI, the presence or absence of the following was also analyzed: hyperintensity on FLAIR, diffusion restriction on DWI, hemorrhage on SWI, susceptibility vessel sign on SWI, empty delta sign on contrast-enhanced T1WI, multifocality, and atypical distribution against arterial territory. To evaluate the location of bright sinus appearance relative to the site of cerebral venous thrombosis, a 3D localization tool available on the picture archiving and communication system was used.

For patients with arterial occlusion, following MR features were analyzed: bright signal intensity in occluded arterial segment on ASL, bright signal intensity in dural sinuses, hyperintensity on FLAIR, diffusion restriction on DWI, hemorrhage on SWI, susceptibility vessel sign on SWI, and multifocality.

### 2.4 Statistical analysis

All statistical analyses were performed using statistical software MedCalc, version 11.1.1.0 (MedCalc, Mariakerke, Belgium). McNemar 2-tailed test was used to compare the sensitivities of bright sinus appearance and findings based on other conventional MR images for the identification of cerebral venous thrombosis. The difference in CBF values between hypoperfused brain parenchyma and contralateral brain parenchyma was evaluated with independent *t* test. Chi-square or Fisher's exact test was used to find differences in MR features of acute ischemic stroke from cerebral venous thrombosis. Linear κ coefficients were calculated to assess the interobserver agreement between 2 readers regarding the presence of bright sinus appearance and hypoperfusion in brain parenchyma on ASL, and susceptibility vessel sign, empty delta sign, and atypical distribution against arterial territory on conventional MR images. Statistical significance was considered when *P* value was less than .05.

## 3 Results

Clinical characteristics and imaging findings of patients are summarized in Table 1.

**Table 1:**
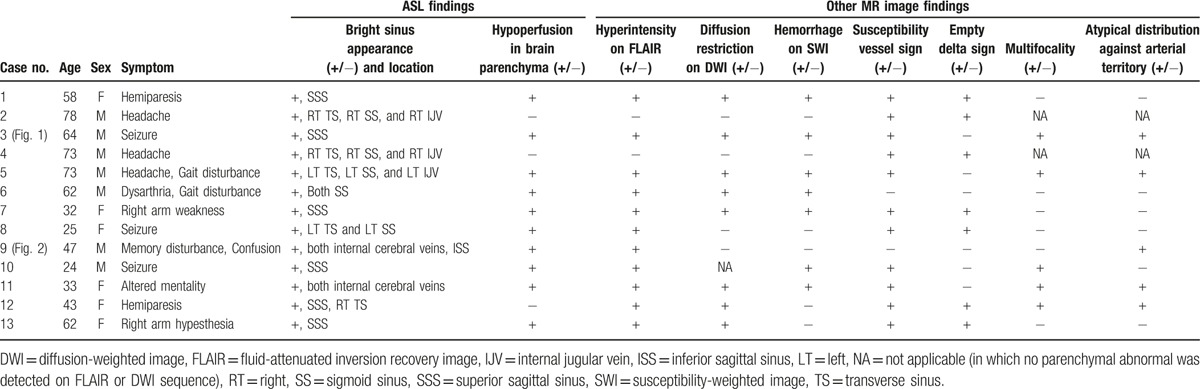
Clinical characteristics and imaging findings of patients.

ASL bright sinus appearance was found in all (100%) 13 patients. In addition, 10 (77%) of the 13 patients showed hypoperfusion in the brain parenchyma drained by the thrombosed sinus on ASL-PWI. CBF value was significantly lower for hypoperfused brain parenchyma than for contralateral normal parenchyma (mean, 23.3 mL/min/100 g; interquartile range, 15.31–31.29 mL/min/100 g vs mean, 43.43 mL/min/100 g; interquartile range, 27.63–59.22 mL/min/100 g, *P* = .019). Mean relative CBF value was 0.58 with interquartile range of 0.18 to 0.77.

FLAIR hyperintensity and diffusion restriction on DWI in the brain parenchyma were seen in 11 (85%) and 8 (62%) patients, respectively. All 10 patients with hypoperfusion in the brain parenchyma drained by the thrombosed sinus on ASL-PWI also showed abnormal signal intensity on the parenchymal abnormality on FLAIR and/or DWI. In contrast, 3 patients without brain parenchymal signal change failed to show any perfusion change on ASL-PWI.

Susceptibility vessel sign and empty delta sign were revealed in 11 (85%) and 7 (54%) patients, respectively. Atypical distribution against arterial territory was seen in 5 (50%) of the 10 patients with parenchymal abnormality on FLAIR and/or DWI. Therefore, the bright sinus appearance had higher sensitivities for identifying cerebral venous thrombosis than the susceptibility vessel sign, empty delta sign, and atypical distribution against arterial territory (with differences of 15%; *P* = .500, 46%; *P* = .031 and 50%; *P* = .031, respectively).

There were perfect interobserver agreements between the 2 readers for both bright sinus appearance and hypoperfusion in brain parenchyma on ASL for both [*κ* = 1.00; 95% confidence interval (95% CI), 1.00–1.00, for both]. In addition, for susceptibility vessel sign, empty delta sign, and atypical distribution against arterial territory on conventional MR images, interobserver agreements were substantial (*κ* = 0.61; 95% CI, 0.00–1.00), substantial (*κ* = 0.77; 95% CI, 0.35–1.00), and perfect (*κ* = 1.00; 95% CI, 1.00–1.00), respectively.

**Figure 1:**
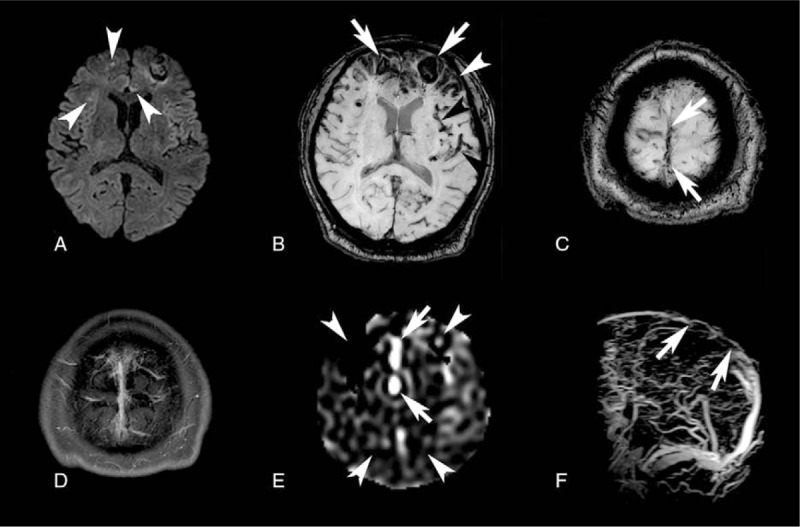
A 64-year-old man presented with seizure. (A) Diffusion-weighted image reveals a few foci with diffusion restriction (arrowheads). (B) Acute hemorrhages are noted in the bilateral frontal lobes on susceptibility-weighted image (arrows). Hemorrhages along the cortex (white arrowhead) and dark signals along the vessels suggestive of venous thrombus (black arrowheads) are also shown. (C) Susceptibility vessel sign is shown in the superior sagittal sinus on susceptibility-weighted image (arrows). (D) Empty delta sign is not definite on contrast-enhanced T1-weighed image. (E) On arterial spin labeling perfusion-weighted image, bright sinus appearance is apparent in the superior sagittal sinus at proximal to the thrombus-filled portion (arrows). Hypoperfusion in the bilateral cerebral hemispheres is also noted (arrowheads). (F) MR venography reveals the filling defect in the superior sagittal sinus suggesting dural sinus thrombosis (between arrows).

**Figure 2:**
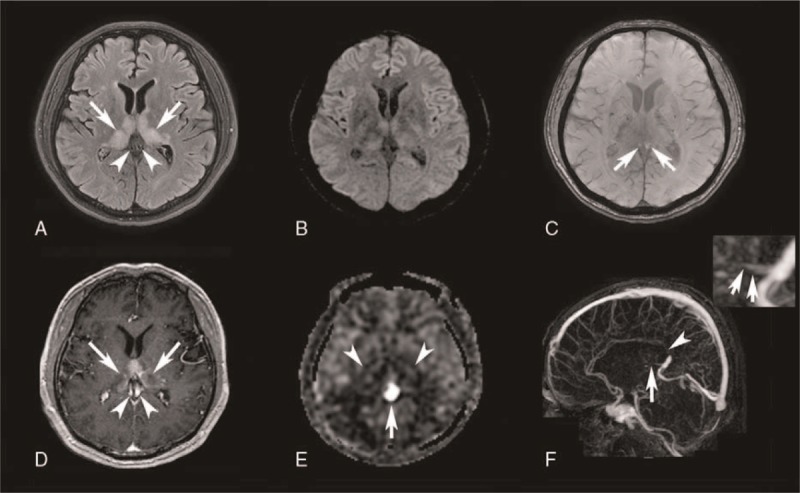
A 47-year-old man presented with memory disturbance and confusion for 2 weeks. (A) Fluid-attenuated inversion recovery image demonstrates hyperintense lesions in the bilateral thalami (arrows) and preserved signal void in the internal cerebral veins implying inner blood flow (arrowheads). (B) No definite abnormal focus with diffusion restriction is noted on diffusion-weighted image. (C) Susceptibility vessel sign is not on susceptibility-weighted image (arrows). (D) Contrast-enhanced T1-weighed image shows enhancement in the bilateral thalami suggestive of subacute stage infarct (arrows). However, empty delta sign in the internal cerebral veins is not definite (arrowheads). (E) On arterial spin labeling image, bright sinus appearance is apparent at internal cerebral veins (arrow). Hypoperfusion in the bilateral thalami is also noted (arrowheads). Bright sinus appearance was also apparent at inferior sagittal sinus (not shown). (F) Four-dimensional CT venography revealing filling defects in the bilateral internal cerebral veins at distal portions (arrow in the raw image and arrows in the zoomed image). Another filling defect is noted at inferior sagittal sinus at distal portion (arrowhead).

Representative MR images including ASL-PWI are shown in Figs. 1 and 2.

In the case of arterial occlusion, 19 (95%) of the 20 patients showed ASL bright vessel appearance in occluded arterial segment, although no patients with cerebral venous thrombosis demonstrated bright signal intensity in artery (*P* < .001). None of the patient accompanied bright sinus appearance, while all patients with venous thrombosis did (0% vs 100%, *P* < .001). FLAIR hyperintensity and diffusion restriction on DWI were observed in 16 (80%) and 17 (85%) patients. Hemorrhage and susceptibility vessel sign at the occluded artery on SWI were seen in 1 (5%) and 17 (85%) patients. Patients with arterial occlusion had significantly less hemorrhage on SWI compared with patients with cerebral venous thrombosis (5%; 1/20 vs 54%; 7/13, *P* = .003). However, presence of other MRI features such as FLAIR hyperintensity, diffusion restriction on DWI, susceptibility vessel sign on SWI, and multifocality was not significantly different between 2 groups.

## 4 Discussion

The pathophysiology of cerebral venous infarction differs from that of arterial ischemic stroke. Cerebral venous thrombosis results in an increased venous pressure that can lower cerebral perfusion pressure and induce parenchymal change due to vasogenic edema, cytotoxic edema, or intracranial hemorrhage.^[14,15]^ Parenchymal change in cerebral venous thrombosis is potentially reversible with appropriate treatment before permanent brain damage occurs.^[14,15]^ Thus, prompt and accurate diagnosis is of great clinical importance.

We hypothesized that, despite signal weakening by post-labeling decay within venous structures, sluggish or arrested blood flow in the dural sinus proximal to thrombus might demonstrate bright signal intensity due to excessive accumulation of labeled protons. This mechanism is similar to that for the bright vessel appearance previously reported in cases of arterial occlusion in acute ischemic stroke or brain death, except that post-labeling decay is not of concern in these settings.^[12,13]^ In our study population with arterial occlusion, the sensitivity of bright vessel appearance in occluded arterial segment was 95%, which is consistent with previous study.^[12]^ Similarly, the sensitivity of bright sinus appearance for identifying the cerebral venous thrombosis using CT or MR venography (with/without cerebral angiography) as the reference standard was 100%. In addition, the sensitivity of bright sinus appearance was significantly higher than that of empty delta sign (54%) or atypical distribution against arterial territory (50%). Therefore, the bright signal intensity in dural sinus could be used to facilitate the detection of cerebral venous thrombosis. However, careful interpretation is required because bright signal in the dural sinus has been reported in patients without cerebral venous thrombosis due to direct passage of the labeled flow from artery to vein such as arteriovenous malformation and sole arteriovenous fistula.^[16]^

We also hypothesized that decreased cerebral perfusion pressure could appear as hypoperfusion of brain parenchyma on ASL-PWI because a previous study reported that cerebral venous thrombosis patients showed decreased CBF on perfusion-weighted imaging.^[14]^ As hypothesized, most patients showed hypoperfusion in the brain parenchyma drained by the thrombosed sinus on ASL-PWI. All these patients also showed abnormal signal intensity on the parenchymal abnormality on FLAIR and/or DWI. In contrast, patients without brain parenchymal signal change failed to show any perfusion change on ASL-PWI. These results imply that patients without brain parenchymal change might be at the early stage of venous occlusion in which compliant venous bed dilatation maintains venous pressure and cerebral perfusion.

ASL-PWI is currently more widely used in routine clinical settings due to its noninvasiveness nature without injecting exogenous contrast medium with absolute quantification possible for the CBF value.^[17,18]^ With improvements in imaging technique such as background suppression and image readout, its signal intensity-to-noise ratio has been increased, while susceptibility artifact and motion sensitivity have been decreased.^[16,18]^ In addition, ASL-PWI can be easily embedded into routine MR sequences or specific MR sequences for patients who are suspected of venous infarct with cerebral venous thrombosis. Therefore, ASL-PWI might be used as a feasible and noninvasive method to evaluate the presence of cerebral venous thrombosis and secondary change of cerebral perfusion.

Our study had a few limitations. First, even though patients from 7 years have been included in the present study, the number of patients with cerebral venous thrombosis was still small presumably due to the rarity of cerebral venous thrombosis in patients and the strict inclusion criteria applied in the present study. Second, we visually assessed perfusion change or bright signal intensity on ASL-PWI. Although it was not an objective method, it seems to be more feasible to use such a method in routine clinical setting than using a quantitative measurement. In addition, we measured absolute CBF values in the hypoperfused brain parenchyma to overcome the subjectivity of visual assessment. CBF value was significantly lower for hypoperfused brain parenchyma than for contralateral normal parenchyma. Finally, the final diagnoses of cerebral venous thrombosis were determined on the basis of neurologic symptoms suspected of cerebral venous thrombosis and CT or MR venography (with/without cerebral angiography). These results might not always coincide with true cerebral venous thrombosis. In some patients, images might not be useful for the detection of small thrombus.

In conclusion, bright sinus appearance on ASL-PWI can be used as an important diagnostic clue for the identification of cerebral venous thrombosis and this technique may have the potential to be used as a noninvasive diagnostic tool to identify the cerebral venous thrombosis.
